# Fatal Cynanche

**Published:** 1831-01-01

**Authors:** 


### XVII.

### WESTMINSTER HOSPITAL.

### FATAL CYNANCHE.

A CASE, under the term of "fatal erysipelas" has lately been reported from the above hospital, but which appears to have been an inflammation commencing about the fauces, producing external inflammation of the face and neck, and ultimately destroying life by disorganization of the larynx.

The patient was a gentleman's valet, aged 27—and like too many of that tribe, addicted to intemperance. He had got wet feet a few weeks previous to his admission (31st March) but felt no inconvenience till within a week of the above date, when his throat became sore, and, on the 29th March, he found it swelled externally under the base of the lower jaw, on the right side—the swelling and redness extending to the ear, the cheek, and the neck. This was the state of the patient on admission. The difficulty of deglutition was considerable — the surface was hot — pulse 110, soft and oppressed—tongue much furred—bowels had been freely opened. An emetic, consisting of six grains of tartrite of antimony and a scruple of ipecacuanha (a large dose, and as we think, an error in the print) was immediately exhibited, after which ten grains of calomel, "to act freely on the bowels," to be followed up by the *liq. ammon. acet.* Dr. Roe ordered twelve leeches to the external fauces—warm fomentations—calomel and jalap. [APRIL 1.] Very low—pulse 120—vomits frequently—the external redness of a paler hue—passed a restless and delirious night — was purged by the powder. To take a tea-spoonful of brandy occasionally—a mixture of decoct. cinchonæ, confect. aromat. and spt. amm. aromat. In the evening the pulse was 140—deglutition very difficult—neck more swelled. A blister to the neck — brandy to be continued. [2d April.] All the symptoms worse—pupils dilated—voice almost extinct—swallowing nearly impracticable—great discharge of muco-purulent matter from the eyes and nose—tongue dry, chopped, and brown—vomiting somewhat subsided. Calomel and rhubarb to open the bowels. After the operation of the powder, the symptoms appeared to be much improved. The pulse fell to 100, and was much firmer and fuller;—but the difficulty of swallowing remained. [April 3d.] Delirious in the night—the external inflammation has quite disappeared without vesication—internal fauces more affected—pulse 136, very feeble—is delirious at times. A blister to the nape of the neck—ten grains of calomel. In the afternoon the physician ordered him into a warm bath, and to take half an ounce of the decoct. aloes comp. every hour till the bowels are opened. We cannot compliment the accuracy of the reporter in the following prescription.

℞. Ammon. carb. ℥j.

Aq. menth. . ℥vj. M. ft. *haustus* cujus capiat, ℥ss. 4tis horis.

The doses of carbonate of ammonia in this mixture were pretty strong (24 grains each) for an inflamed surface.

"When the ammonia draught was administered, the patient was almost thrown into convulsions, by the sufferings which the attempt at swallowing so strong a stimulant excited." The ammonia mixture was therefore diluted, and the patient got some of it down. The vital forces, however, continued to fail; and in spite of brandy, laudanum, &c. conveyed into the stomach through a tube, the unfortunate patient sunk, and expired early next morning.

We are informed that the patient had formerly suffered much from syphilis, both in its primary and secondary stages, and had been in the habit of taking opium freely. The dissection is passed over in a very unsatisfactory manner, in the following words.

"The post-mortem examination exhibited a highly diseased state of the larynx, especially at its upper parts, the natural characters of the glottis, epiglottis, and chordæ vocales, being nearly obliterated. A high state of inflammation existed in the pharynx and internal fauces."—*Lancet*.

From the symptoms and the dissection, then, we must demur to the title of the disease—"fatal erysipelas." This malady has enough to answer for, without bearing the blame of what may sometimes be done by cynanche laryngea, and c. pharyngea, of which the above patient died.* It might admit of some question whether the external inflammation, characterized as erysipelas, together with the depressed state of the constitutional powers, did not, in the above case, lead the practitioners too far from the local inflammation about the larynx and fauces, which was working the fatal mischief. It is doubtful whether brandy and ammonia will ever remove that debility which results from a disorganizing local process. The most efficient means of restoring the general strength is by reducing the topical inflammation.

* Some of our own reporters are not free from the sin of misnomering. Thus, in our last number, page 543, under the head of "Westminster Hospital," there is a case of fatal "ulceration of the tonsils," in which no disease nor symptom of disease of the tonsils was exhibited before or after death. The case is interesting, though falsely titled. The patient died of disease of the larynx.—*Ed.*

---

